# Creating an interdisciplinary, modular, and policy-oriented county-level mental health index for the United States

**DOI:** 10.3389/fpubh.2026.1887600

**Published:** 2026-07-30

**Authors:** Jared Datzman, Gabriel Allen-Moi

**Affiliations:** 1 Department of Public Health, Des Moines University, Des Moines, IA, United States; 2 Department of Economics, University of Washington, Seattle, WA, United States

**Keywords:** GIS—geographic information system, mental health index, mental health surveillance, principal component analysis—PCA, public mental health (PMH)

## Abstract

**Background:**

In the United States, there is an increased focus on mental health problems and the need for effective estimates of localization of mental health problems is manifest. This study draws on behavioral, economic, social, and public health risk factors to predict county-level mental health for counties in the United States.

**Methods:**

Candidate predictors were identified from a literature review. Selected variables were condensed into relevant submodules and a mental health outcomes index using separate principal component analyses. The submodules were then regressed to predict the mental health index. County-level vulnerability scores were calculated for each predictor to assess their relative importance as suggested by the model.

**Findings:**

A systematic search of available data yielded a set of 56 predictive factors, including novel predictors. Seventeen submodules in five policy domains were derived, explaining 85.1% of the mental health index variance. Tableau was utilized to create interactive maps displaying the submodules and mental health outcomes over counties in the United States for use by policymakers and stakeholders.

**Interpretation:**

This model may be used for several purposes throughout the government and academia, the timeliest of which would be providing information to redirect mental health interventions to maximize outcomes as funders begin pursuing efficiency of spending over volume spent. By utilizing the index and submodules, policymakers can focus their mental health improvement efforts in the most impactful areas for their constituencies.

## Introduction

### The public health burden of mental illness

The United States has recently experienced a renewed interest in evaluating the effectiveness and efficiency of government agencies and recalcitrantly rising rates of mental health issues in the population. In light of this situation, a simple method of checking in on public mental health and its determinants has become more desirable than ever. Addressing this need would provide governments with greater insights into the performance of agencies, policies, and the impact of recent shocks to society, allowing non-governmental organizations to track areas in need of mental health services.

The American mental health treatment industry is valued at approximately $96.9 billion in 2025 and is projected to grow to $151.6 billion in the forthcoming decade, without considering unmeasured impacts of subclinical distress, untreated individuals, or secondary market impacts such as those on the insurance industry (1). Approximately 23.1% of American adults are affected by a clinically significant mental illness, as of 2022 (2). Despite aggressive interventions and the advent of remote treatment technologies, the incidence of a major depressive episode, for example, increased by 27%–122% for those younger than 30 in the United States from 2005 to 2017 (3). In 2023, 1.5 million individuals in the United States have attempted suicide, and 14.21 persons per 100,000 Americans completed the attempt (4). There has been extensive academic and government interest in the establishment of a similar apparatus in the United States (5–7).

### Mental health surveillance: global context

Public health surveillance systems have become an increasingly important tool for policymakers in the wake of the coronavirus disease 2019 (COVID-19) pandemic, as they allow agents to know where problems are so they can reallocate resources to combat them efficiently. Recently, increasing administrative pressures for efficiency over the volume of dollars spent have redoubled the urgency of developing these tools. Mental health surveillance (MHS) systems, when disaggregated from general health surveillance systems, are designed to assist local agents and national organizations in allocating resources more efficiently (5). Many countries are still exploring this relatively novel frontier, with only 13% of the responding WHO member nations asserting that they had reports on the last 2 years of mental health data they had compiled in 2020. Only 55% of the responding WHO member states reported using metrics to inform and monitor their mental health policies, despite 86% of member states having an integrated, independent policy or program on mental health (8). A search of PubMed and Academic Search Complete revealed that countries such as China, Canada, Australia, Switzerland, and Germany have already undertaken the development of a fully independent and specialized MHS apparatus ahead of their peers (9). One of the most notable examples is the MHS system operated by the Robert Koch Institute (RKI) in Germany, which uses a systematic, continuous monitoring approach to track the wellbeing of the population at a national level, enabling rapid identification of trends and support healthcare planning (10).

### Existing surveillance infrastructure in the United States

Despite the pressures of an increasingly severe mental health crisis, a comparable framework for public health surveillance as applied to mental health has yet to emerge in the United States. A review of the literature revealed that within the United States, efforts in similar projects have been only limited, yielding small or untested degrees of predictability, often tailored to the needs of a demographic of interest, or addressing geographical units of organization that are unwieldy and difficult to dissolve into larger units or break down into smaller units. Various departments of the federal government have collected region- and population-level mental health metrics, such as those included in the CDC’s Behavioral Risk Factor Surveillance System (BRFSS). However, these data have remained dispersed across different programs conducted by multiple departments rather than constituting an integrated surveillance apparatus. Currently, county-level data are available in limited formats: the CDC’s Population-Level Analysis and Community Estimates (PLACES) program, which produces model-based county-level estimates from BRFSS via small-area estimation (11); the County Health Rankings and Roadmaps program, which aggregates county-level data from a variety of sources and produces annual rankings on health factors and outcomes that include several mental health-relevant indicators (12); and the CDC/ATSDR Social Vulnerability Index (SVI), which identifies socially vulnerable populations for disaster and emergency preparedness planning (13).

### Gaps in existing approaches

These programs represent genuine contributions to county-level health surveillance; however, the authors have identified a gap in the existing literature in that these instruments focus on indicators with a generalized outlook rather than being empirically weighted toward mental health outcomes. While the PLACES program and county health rankings inform policymakers about the prevalence of mental ill-health in a county, they do not provide a parallel, empirically weighted framework for understanding the drivers most closely associated with those outcomes. As a result, these tools cannot readily indicate whether a county should prioritize economic, social, physical health, or environmental factors based solely on the index data. The SVI was designed for emergency preparedness rather than MHS and was never validated against a measured mental health outcome. Relying on any single survey or metric when estimating a target variable can introduce bias and increase uncertainty due to variable’s unique characteristics, making it beneficial to incorporate multiple sources of information into county-level estimations. Key measures of mental health are gathered from the National Health Interview Survey (NHIS), the National Survey on Drug Use and Health (NSDUH), and other nationally representative instruments; however, these measures focus only on mental health as an outcome, without identifying county-level factors that may indicate the reason for poor mental health in those areas. The evidence reviewed here suggests that, although the data infrastructure necessary to construct a strongly predictive, county-level mental health index already exists in the United States, there is currently no instrument that has organized and empirically weighted these data specifically for mental health outcomes. Such an instrument would ideally include county-level submodules designed to identify important factors contributing to poor mental health in each respective county.

### The present study

To address the issues noted above, this study developed a MHS framework for the United States that incorporates a wide range of predictors drawn from global precedents and studies focused on identifying individual determinants of mental health. These predictors were distilled into a smaller number of submodules and organized into modules reflecting their relevant policy domains, for maximum legibility to those without a specialized mental or public health background. Submodule and indicator-level scores were then empirically weighted against measured county-level mental health outcomes rather than assigned by expert judgment alone. The resulting index can be assigned a value for each county in the United States, with the modular structure allowing a policymaker to identify not only where mental health is poor but which domains and specific indicators are driving that result. The aim of this study is, therefore, to develop a system of indices that can predict county-level mental health problems and potential vectors of mental health vulnerability for all counties within the United States using publicly available county-level data, and to demonstrate that by weighting predictors directly on their ability to forecast mental health outcomes. It is possible to provide indices that are more empirically grounded and more directly actionable than those currently available.

## Methods

An index design was selected because it would allow for the organization of predictors into intuitive modules and submodules that would make relevant information readily available to appropriate stakeholders. Previous literature suggests that a public health surveillance program ought to have timely, regularly updated releases in order to keep local institutions abreast of the effects of current events and trends (14). Thus, measures were selected based on the regular release and availability of the data with the primary vintage of the data aimed at the years of 2022–2024.

In addition to being measured at a county level and updated on a regular basis, indicators were curated on based on a number of criteria synthesized from recommendations in the literature (15, 16).

- Face validity: The relevance and effect of the indicator was readily apparent and plausibly affected the target variables. It appears to measure what it was intended to.
- Precedent: The measure has been used to predict the target variable, its components, or its close correlates in the past.
- Policy orientation: Has a clear association with one or more policy domains or specific policies, not likely to be perversely misinterpreted or gamed by policymakers.
- Reliability: Measures can be replicated given identical conditions.
- Generalizability: Measures can be employed regardless of contemporary events and are not predicated upon a shock or temporary phenomenon.
- Legibility: A member of the intended audience for the model can understand what the indicator is and why it is relevant.
- Agency: The model focuses on metrics which are under the control of key agents and decision makers.

Data were gathered from publicly available sources as detailed in Supplementary Table 1A; these data were drawn from literature regarding the determinants of mental health and separated into six modules: Demographic, Economic, Physical Health, Environmental, Social, and Mental Health Outcomes. These modules function as an intuitive framework, and predictors were organized into these modules for enhancing the legibility of the model to non-specialist end users.

Six calculated variables were created for this analysis: a measure of ethnic diversity, population growth volatility, income volatility, income growth volatility, unemployment volatility, and production volatility. Ethnic diversity was calculated by subtracting the largest ethnic group in the county from 100; for example, if the largest ethnic group in a county was non-Hispanic White, accounting for 73% of the population according to census data, the diversity measure would be 0.27. All volatilities were operationalized as the standard deviation of the metric over the last 5 years of the most recent data.

All variables were standardized prior to use in subsequent analyses with skewed variables being log-transformed before standardization to allow for better performance in the principal component analyses. Multiple imputation by chained equations (MICE) using weighted predictive mean matching was utilized to address missing variables, and two separate MICE analyses were run: one on potential predictors and one on potential outcomes (17, 18). While it is not standard to use MICE to address missing outcome variables, this was deemed necessary for the stated goal of the project to estimate a mental health index for all US counties. This approach avoids introducing artificial associations between the predictor and outcome variables, since the two analyses used entirely separate variable sets: one included only the variables measuring the outcomes of interest, and the other included only the variables measuring the predictors. The MICE analyses were enhanced by incorporating potential predictors (e.g., the percent of the population with insurance) and potential outcomes (e.g., Mental Health America’s Depression estimates) that ended up not being used due to superfluousness or failure of the variable to meet the above criteria (e.g., regular updates), see Supplementary Table 1A for all variables used, including those used in the MICE analyses, which were not included in the final modelling.

### Analysis

All variables were first sorted into predictor and outcome groupings. One outcome grouping consisting of variables related to mental health in a county was created. Predictors were organized from five modules noted above into 17 predictor groupings: diversity of the population, age and total population, economic inequality, poverty, economic volatility, unemployment metrics, weather, indicators of a disrupted family life, institutions aimed at strengthening family life, social capital, access to community resources, social isolation, indicators of antisocial behavior, indicators of physical health and wellbeing, measures of health-related behaviors, measures of physical disability, and measures of mortality.

**Figure 1:**
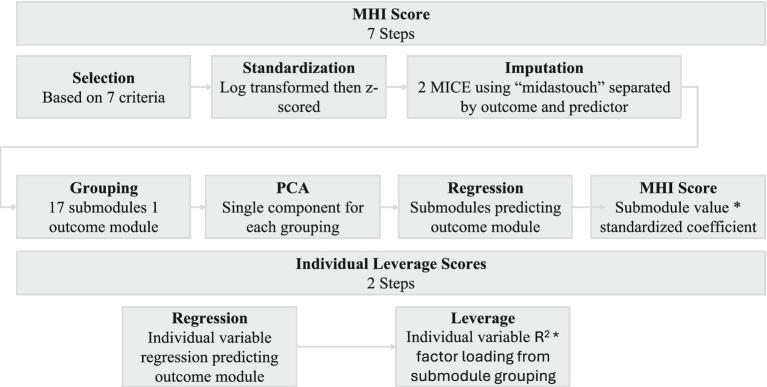
Analysis flow chart.

The predictor and outcome groupings noted above were utilized in single measure principal component analyses to derive a metric for each index. The submodules derived from this analysis were then used in a regression predicting the outcome module. Each submodule score was then multiplied by standardized coefficient from the prior regression and summed to create an overall mental health index score. Finally, each submodule was then used in a regression to predict the mental health index to create an individualized *R*^2^ which was multiplied by the factor loadings from the principal component analysis to create a mental health leverage score for each predictor. All principal component analyses and regressions were performed in Python. Figure 1 shows the steps performed for the analyses.

## Results

### Model performance

A regression predicting the mental health index using all 17 submodules yielded a significant association explaining 85.1% of the variance in the outcome variable (*F* = 1,049.49, *p* < 0.0001) with a root mean square error of 0.763. Table 1 shows the significance level and standardized betas for each submodule in the model.

**Table 1:** Regression coefficients.

Submodule	Standardized coefficient (*β*)	*p*-value
(Intercept)	>0.001	>0.999
Diversity	−0.085	<0.001
Age	−0.006	0.5661
Inequality	0.042	<0.001
Economic volatility	−0.064	<0.001
Poverty	−0.018	0.0617
Unemployment	0.12	<0.001
Temperature	−0.059	<0.001
Disrupted family	−0.021	0.014
Isolation	0.052	<0.001
Family institutions	−0.027	0.002
Social capital	−0.092	<0.001
Access	−0.101	<0.001
Antisociality	0.05	<0.001
Physical health	0.82	<0.001
Physical disability	0.088	<0.001
Health behavior	−0.142	<0.001
Fatality	−0.071	<0.001

The regression coefficients for numeric features are standardized.

Notably the strongest standardized beta was physical health indicating a strong connection between the physical health submodule and the mental health outcome variable. However, multicollinearity makes interpreting the coefficients perilous. To investigate further, Table 2 demonstrates the mean dropout loss for each submodule when predicting the outcome variable and the *R*^2^ change for each submodule. The most important factor by dropout loss and *R*^2^ the mental health index was the physical health submodule.

**Table 2:** Model fit predicting mental health index.

Submodule (module)	Mean dropout loss	Module	*R*^2^ change
Physical health (health)	1.987	Health	0.815
Arthritis		Social	0.011
Average number of unhealthy days		Economic	0.014
Chronic obstructive pulmonary disorder rate		Environment	0.008
High blood pressure rate		Demographics	0.003
High cholesterol rate			
Previous hospitalization rate			
Unemployment (economic)	0.683		
Unemployment rate			
Number of establishments			
Labor force participation			
Disability (health)	0.681		
Vision disability			
Self-care disability			
Hearing disability			
Mobility disability			
Diversity (demographic)	0.669		
Foreign born			
Recent arrival (domestic origin)			
Recent arrival (foreign origin)			
Ethnic diversity			
Lack of access (environmental)	0.658		
Lack of reliable transport			
Internet access			
Park accessibility			
Mental healthcare accessibility			
Social capital (social)	0.656		
Census mail-back rate			
Voter participation rate			
Social association rate			
Economic volatility (economic)	0.642		
Population growth volatility			
Income volatility			
Income growth volatility			
Unemployment volatility			
Production volatility			
Health behaviors (health)	0.637		
Physical inactivity			
Smoking			
Adult obesity rate			
Excessive drinking rate			
Sexually transmitted disease/infection rate			
Human immunodeficiency virus rate			
Fatality (health)	0.636		
Life expectancy			
Drug and alcohol-related mortality			
Temperature (environmental)	0.633		
Maximum temperature			
Minimum temperature			
Average temperature			
Sunshine			
Particulate matter (2.5 μm)			
Inequality (economic)	0.627		
Food insecurity			
Price parity			
Gini coefficient			
Antisociality (social)	0.626		
Average homelessness rate			
Adult arrests			
Family institutions (social)	0.625		
Number of religious institutions			
Percent married			
Nursery preschool enrollment			
Disrupted families (social)	0.624		
Percent single male parent households			
Percent single female households			
Juvenile arrests			
Isolation (social)	0.623		
Feelings of social isolation			
Lack of social and emotional support			
No leisure-time activity			
Poverty (economic)	0.622		
Gross domestic product per capita			
Living expense as percent of paycheck			
Poverty rate			
Age (demographic)	0.622		
Percent population aged 30–40			
Percent population aged 65+			
Percent population aged 14–24			
Total population			

### Predictor performance

Table 3 shows the principal component analysis eigenvalues, loadings for each variable used in the submodules, the individual regression *R*^2^ for each submodule included in the model, and the resultant mental health leverage score indicating an upper end of impact on mental health that could be achieved by improving upon that variable in a county. The eigenvalues indicate that the physical health variable had the tightest grouping while family institutions had the weakest grouping. In agreement with the model dropout loss physical health had the greatest *R*^2^ while age had the lowest which contributes to the strong differences in vulnerability scores (where higher values indicate a stronger connection of each individual variable to mental health outcomes).

**Table 3:** Loadings, *R*^2^, and leverage of predictors in submodules.

Submodule (module)	Eigenvalue (% variance explained)	Submodule loading	Adjusted *R*^2^	County vulnerability scores
Diversity (demographic)	1.812 (45.3%)		0.029	
Foreign born		0.822		−0.024
Recent arrival (domestic origin)		0.426		−0.012
Recent arrival (foreign origin)		0.671		−0.019
Ethnic diversity		0.711		−0.021
Age (demographic)	2.081 (52.0%)		<0.001	
Percent population aged 30–40		−0.653		−0.001
Percent population percent aged 65+		0.901		0.001
Percent population aged 14–24		−0.635		−0.001
Total population		−0.663		−0.001
Inequality (Economic)	1.243 (41.5%)		0.382	
Food insecurity		0.785		0.300
Price parity		−0.567		−0.217
Gini coefficient		0.554		0.212
Economic volatility (economic)	1.895 (37.9%)		0.051	
Population growth volatility		0.722		−0.037
Income volatility		0.602		−0.031
Income growth volatility		0.614		−0.031
Unemployment volatility		−0.671		0.034
Production volatility		0.429		−0.022
Unemployment (economic)	1.536 (57.5%)		0.175	
Unemployment rate		0.803		0.141
Number of establishments		−0.513		−0.09
Labor force participation		−0.792		−0.139
Poverty (economic)	1.724 (57.5%)		0.102	
Gross domestic product per capita		−0.675		−0.069
Living expense as percent of paycheck		0.825		0.084
Poverty rate		0.767		0.078
Temperature (environmental)	3.648 (73.0%)		0.191	
Maximum temperature		0.965		0.184
Minimum temperature		0.964		0.184
Average temperature		0.976		0.186
Sunshine		0.652		0.125
Particulate matter (2.5 μm)		0.641		0.122
Lack of access (environmental)	1.497 (37.4%)		0.294	
Lack of reliable transport		0.661		−0.194
Internet access		−0.648		0.191
Park accessibility		−0.577		0.170
Mental healthcare accessibility		−0.555		0.163
Disrupted families (social)	1.140 (38.0%)		0.011	
Percent single male parent households		0.568		0.006
Percent single female households		0.665		0.007
Juvenile arrests		0.613		0.007
Social capital (social)	1.254 (41.8%)		0.362	
Census mail-back rate		0.646		−0.234
Voter participation rate		0.816		−0.295
Social association rate		0.409		−0.148
Family institutions (social)	1.059 (35.3%)		0.092	
Number of religious institutions		0.674		−0.062
Percent married		0.642		−0.059
Nursery preschool enrollment		0.439		−0.04
Isolation (social)	2.030 (67.7%)		0.347	
Feelings of social isolation		0.895		0.311
Lack of social and emotional support		0.713		0.247
No leisure-time activity		0.849		0.295
Antisociality (social)	1.142 (57.1%)		0.001	
Average homelessness rate		0.756		−0.001
Adult arrests		0.756		−0.001
Physical health (health)	4.094 (68.2%)		0.803	
Arthritis		0.861		0.691
Average number of unhealthy days		0.902		0.724
Chronic obstructive pulmonary disorder rate		0.922		0.740
High blood pressure rate		0.890		0.715
High cholesterol rate		0.733		0.589
Previous hospitalization rate		0.600		0.482
Health behaviors (health)	3.143 (52.4%)		0.566	
Physical inactivity		0.934		0.529
Smoking		0.852		0.482
Adult obesity rate		0.829		0.469
Excessive drinking rate		−0.682		−0.386
Sexually transmitted disease/infection rate		0.529		0.299
Human immunodeficiency virus rate		0.332		0.188
Fatality (health)	1.634 (81.7%)		0.456	
Life expectancy		0.904		−0.412
Drug and alcohol-related mortality		−0.904		0.412
Disability (health)	3.366 (84.2%)		0.542	
Vision disability		0.961		0.521
Self-care disability		0.971		0.526
Hearing disability		0.760		0.412
Mobility disability		0.961		0.521
Outcome variable (target)	3.881 (64.7%)			
Frequent mental distress		0.977		
Cognitive disability		0.924		
Sleep disruptions		0.801		
Depression		0.756		
Suicide		0.077		

Modules to which each submodule belongs are given in parentheses.

**Figure 2:**
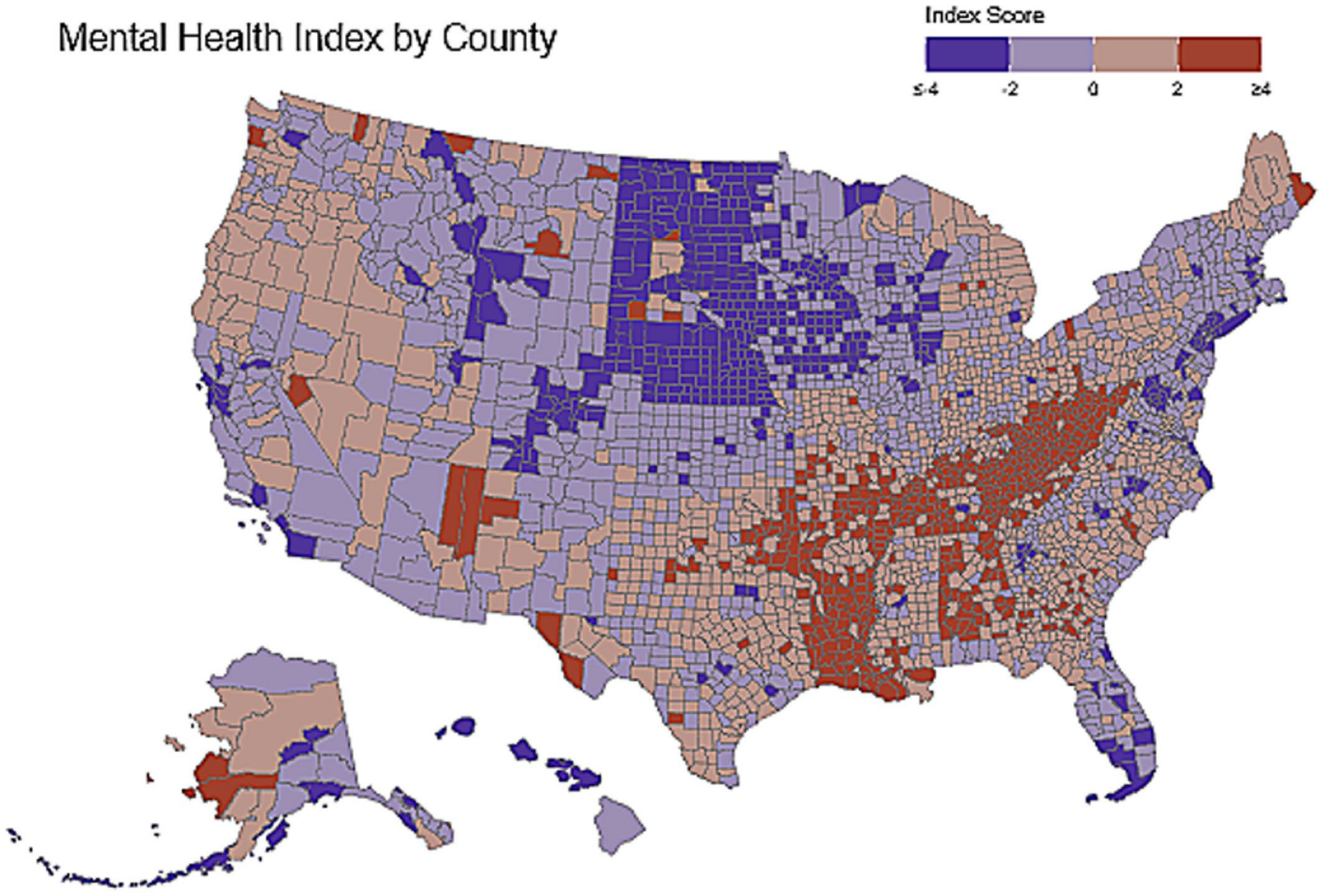
Mental health index across USA counties.

Figure 2 shows the mental health index for each county which highlights several notable trends. Note that larger scores on the index indicate the presence of more mental health problems (in red) and smaller scores indicate the presence of fewer mental health problems (in blue). The mappings of the index and submodules have been posted on a publicly visible tableau dashboard here: https://public.tableau.com/views/FinalDashboard_17699026493970/Story1?:language=en-US&publish=yes&:sid=&:redirect=auth&:display_count=n&:origin=viz_share_link.

## Conclusion

### Discussion of results

Mappings indicate a number of key geographic areas of interest. Counties suffering from worse mental health appear to cluster in the American South, Nevada, and Alaska. Especially notable is the Appalachian areas around Kentucky and Tennessee as well as areas around the Mississippi river in Mississippi and Louisiana. Counties that showed better scores within the index are counties within the Great Plains region, in the American West, Hawaii, and the New England area. Counties with cities appear to generally have better index scores outside of the American South while the index scores for rural areas seem to be more variable. In addition to the major hot spots in the American South a number of counties that demonstrated greater mental health disparities with nearby counties were noted as being tribal lands (e.g., Navajo Nation, Standing Rock Indian Reservation).

Regressions of individual submodules onto the outcome variable reinforce findings from the standardized coefficients in Tables 1, 2. Physical health, disability, and fatality clearly have a profound relationship with mental health, with prevalence of COPD being the predictor with the single greatest vulnerability score. Interestingly however, despite COPD being strongly associated with age, the age submodule’s predictivity was miniscule. This may be due to dilution of the submodule with total population and younger age group. Submodules focused on younger demographics, such as disrupted families and family institutions may have an understated predictivity, as the mental health index focuses on adult mental health issues, if they are predictive at all on the community level. Future refinements of this public health surveillance system should aim to represent children more in the mental health index, while it is advisable that governments collect more detailed information on child mental wellbeing.

Mental healthcare facilities, excessive drinking, as well as the access and volatility submodules, have seemingly paradoxical county vulnerability scores. In the case of mental healthcare facilities, it could be the case that they are simply located where demand is greatest, or the high vulnerability score could be attributable to a reporting bias. The access submodule may be subject to a confounding variable (e.g., rurality) and this could be an avenue for further exploration in the future. The volatility submodules’ low vulnerability score is likely attributable to economic growth being more consistent than year-over-year losses. The ostensibly protective nature of excessive drinking is likely attributable to confounds with age, sociability, and socioeconomic status. For example, college-aged young adults may be healthier physically and mentally than other age groups regardless of drinking, but excessive drinking can still predict poorer mental health within this age group.

The creation of the mental health index allows for geographic, and eventually, temporal patterns and clusters to be identified for mental health alongside each submodule allowing the identification of locally relevant covariate groups for places with elevated mental health problems. This will allow for further research into the nature of the mental health situations for these clusters and in turn may allow for the emergence of novel strategies to redress issues in these specific areas. The current index and submodule structure offers several benefits over current measures such as the SVI or individual measures of the BRFSS. First, it orients measures specifically to mental health by its weighting procedure. Second, it offers numerous submodules which allow for more nuanced investigations into factors connected to a county’s mental health score. Finally, the proposed index includes leverage weights for individual variables which allow for estimations of effectiveness of interventions aimed at the variables.

One limitation of this study concerns the construct validity of each of the submodules. However, this is beyond the scope of the study, as these indicators are not used here to argue for the existence of say, “Family Institutions” as a construct, but simply as an epiphenomenon which predicts the presence of mental health issues in a county. It is nevertheless an important detail to note, as it could prove informative to establish submodule construct validity for use outside of this index. A further limitation is the reliance on the Missing At Random (MAR) assumption required for the MICE analyses used to address missingness in the data, this assumption is tentative due to the tendency for counties with smaller populations to be less well represented in the data.

Another limitation of the study is that this model only measures the predictivity of these factors on negative mental health and mental illness. This means that while capital and inequality may not in and of themselves be predictive of mental health *issues*, they may still be quite predictive of positive mental health and subjective wellbeing. The data used were also collected at the county level from numerous different organizations, surveys, and methods which may introduce extra heterogeneity into the model. Finally, some of the data were collected from separate years/year groupings based on the need for masking, when the data were most recently available, and a desire for accuracy and representativeness for each county.

Despite these limitations the index and submodules provide useful measures which can be readily utilized for further analysis, needs-assessments, and grant-writing support. Further research should focus on searching for natural experiments where discrete shocks to certain predictors are observed in a subset of counties, establishing construct validity and alternative uses for the submodules, forecasting, and longitudinal observations. Applying the index retrospectively to previous years may also prove fruitful in facilitating longitudinal research.

## Data Availability

The original contributions presented in the study are included in the article/Supplementary material, further inquiries can be directed to the corresponding author.

## Supplementary material

SUPPLEMENTARY TABLE S1 Data vintage table.
