# Supplementary material for: Creating an interdisciplinary, modular, and policy-oriented county-level mental health index for the United States
Source: Front Public Health. 2026 Jul 30;14:1887600. doi: 10.3389/fpubh.2026.1887600 (PMC13467919; doi:10.3389/fpubh.2026.1887600)
Supplement: Supplementary file 1 [file Table_1.DOCX]

**Appendix**

**Table 1A.**

| **Predictors** | **Data Vintage** | **Source** |
| --- | --- | --- |
| **Diversity (Demographic)** |  |  |
| Foreign Born | 2023 | ^1^ |
| Recent Arrival (Domestic Origin) | 2023 | ^1^ |
| Recent Arrival (Foreign Origin) | 2023 | ^1^ |
| Ethnic Diversity | 2021^✝^ | ^2^ |
| **Age (Demographic)** |  |  |
| Percent Population Aged 30 to 40 | 2021^✝^ | ^2^ |
| Percent Population Percent Aged 65+ | 2021^✝^ | ^2^ |
| Percent Population Aged 14 to 24 | 2021^✝^ | ^2^ |
| Population | 2023 | ^1^ |
| **Inequality (Economic)** |  |  |
| Food Insecurity | 2022 | ^3^ |
| Price Parity | 2023 | ^4^ |
| Gini Coefficient | 2023* | ^1^ |
| **Economic Volatility (Economic)** |  |  |
| Population Growth Volatility | 2018-2023 | ^1^ |
| Income Volatility | 2018-2023 | ^5^ |
| Income Growth Volatility | 2018-2023 | ^5^ |
| Unemployment Volatility | 2019-2024 | ^5^ |
| Production Volatility | 2018-2023 | ^5^ |
| **Employment (Economic)** |  |  |
| Unemployment Rate | 2023* | ^1^ |
| Number of Establishments | 2022 | ^6^ |
| Labor Force Participation | 2023* | ^1^ |
| **Poverty (Economic)** |  |  |
| Gross Domestic Product Per Capita | 2023 | ^5^ |
| Living Expense as Percent of Paycheck | 2025 | ^7^ |
| Poverty Rate | 2023 | ^8^ |
| **Temperature (Environmental)** |  |  |
| Maximum Temperature | 2024 | ^9^ |
| Minimum Temperature | 2024 | ^9^ |
| Average Temperature | 2024 | ^9^ |
| Sunshine | 2020 | ^10^ |
| Particulate Matter 2.5 | 2019 | ^10^ |
|  |  |  |
| **Access (Environmental)** |  |  |
| Lack of Reliable Transport | 2022 | ^11^ |
| Internet Access | 2023* | ^1^ |
| Park Accessibility | 2024 | ^12^ |
| Mental Healthcare Accessibility | 2024 | ^13^ |
| **Disrupted Families (Social)** |  |  |
| Percent Single Male Parent Households | 2023* | ^1^ |
| Percent Single Female Households | 2023* | ^1^ |
| Juvenile Arrests | 2022 | ^14^ |
| **Social Capital (Social)** |  |  |
| Census Mail-Back Rate | 2020 | ^15^ |
| Voter Participation Rate | 2020 | ^16^ |
| Social Association Rate | 2022 | ^6^ |
| **Family Institutions (Social)** |  |  |
| Number of Religious Institutions | 2022 | ^6^ |
| Percent Married | 2023* | ^1^ |
| Nursery Preschool Enrollment | 2023 | ^1^ |
| **Isolation (Social)** |  |  |
| Feelings of Social Isolation | 2022 | ^11^ |
| Lack of Social and Emotional Support | 2022 | ^11^ |
| No Leisure-Time Activity | 2022 | ^11^ |
| **Antisociality (Social)** |  |  |
| Average Homelessness Rate | 2013-2018 | ^17^ |
| Adult Arrests | 2022 | ^14^ |
| **Physical Health (Health)** |  |  |
| Arthritis | 2022 | ^18^ |
| Chronic Obstructive Pulmonary Disorder Rate | 2022 | ^11^ |
| High Blood Pressure Rate | 2022 | ^11^ |
| High Cholesterol Rate | 2022 | ^11^ |
| Previous Hospitalization Rate | 2022 | ^19^ |
| **Health Behaviors (Health)** |  |  |
| Physical Inactivity | 2023 | ^18^ |
| Smoking | 2023 | ^18^ |
| Adult Obesity Rate | 2023 | ^18^ |
| Excessive Drinking Rate | 2023 | ^18^ |
| Sexually Transmitted Disease Rate | 2023 | ^20^ |
| Human Immunodeficiency Virus Rate | 2024 | ^20^ |
| **Fatality (Health)** |  |  |
| Life Expectancy | 2020-2022 | ^21^ |
| Drug and Alcohol-Related Mortality | 2018-2023 | ^22^ |
| Outcome Variable |  |  |
| Frequent Mental Distress | 2022 | ^11^ |
| Cognitive Disability | 2022 | ^11^ |
| Sleep Disruptions | 2022 | ^11^ |
| Depression | 2022 | ^11^ |
| Web-based Injury Statistics Query and Reporting System | 2022 | ^23^ |
| **Extra Predictors in Multiple Imputation by Chained Equations** |  |  |
| Food environment index | 2021 | ^18^ |
| % Fair or Poor Health | 2021 | ^18^ |
| % Women Married (except separated) Aged 35-44 | 2021 | ^1^ |
| Ratio of Unmarried Men 15 to 44 years per 100 unmarried women 15 to 44 years | 2021 | ^1^ |
| Housing Cost % of Paycheck | 2021 | ^1^ |
| Income to Debt Ratio | 2023 | ^24^ |
| Graduation Rate | 2020-2021 | ^25^ |
| % of population with insurance | 2021 | ^2^ |
| Received food stamps in the past 12 months among adults | 2021 | ^2^ |
| **Extra Outcomes in MICE** |  |  |
| Average number of mentally unhealthy days | 2022 | ^18^ |
| Number of People Scoring with Severe Depression | 2023 | ^26^ |
| Number of People Reporting Frequent Suicidal Ideation | 2023 | ^26^ |
| Number of People Scoring Positive for PTSD | 2023 | ^26^ |
| Number of People Identifying as Trauma Survivors | 2023 | ^26^ |
| Number of People At Risk for Experiencing Psychotic-Like Episodes | 2023 | ^26^ |

*****5-Year ACS estimate for year listed

✝Slightly older data used for consistency due to lack of data on the state of Connecticut

**References:**

1.	Bureau USC. Data from: American community survey (ACS). *Censusgov*. 2023.

2.	Bureau USC. Data from: Age and sex composition in the united states: 2021. *Censusgov*. 2022.

3.	United States Department of Agriculture Economic Research S. Data from: Food environment atlas | economic research service.

4.	Commerce USDo. Estimating county-level regional price parities from public data | U. S. Department of commerce. 2024/03/27/T14:12:41-04:00 2024;

5.	Bureau of Economic A. Data from: BEA data | U. S. Bureau of economic analysis (BEA). 2024.

6.	Bureau USC. Data from: County business patterns. *Censusgov*. 2023.

7.	Economic Policy I. Family Budget Map. *Economic Policy Institute | Family Budget Map*. 2025 2025;

8.	Bureau USC. Data from: SAIPE state and county estimates for 2023. *Censusgov*. 2024.

9.	information NNCfE. Data from: Climate at a Glance: County Mapping. *Climate at a Glance | County Mapping | National Centers of Environmental Information (NCEI)*. 2025.

10.	Cdc. Data from: National environmental public health tracking network data explorer.

11.	Centers for Disease C, National Center for Chronic Disease P, Health P, Division of Population H. Data from: PLACES: Local data for better health, county data 2024 release. 2025.

12.	Trust for Public L. Data from: ParkServe Data Downloads - Trust for Public Lands. *ParkServe Data Downloads - Trust for Public Lands*. 2024.

13.	Center for M, Medicaid S. National provider identifier standard (NPI) | CMS.

14.	United States. Federal Bureau Of I. Data from: Uniform crime reporting program data: arrests by age, sex, and race, united states, 2022: version 1. 2024. doi:10.3886/ICPSR39062.V1

15.	Bureau USC. Data from: Decennial self-reponse rates 2020. *Censusgov*. 2021.

16.	Data MITE, Science L. Data from: County presidential election returns 2000-2024. 2018. doi:10.7910/DVN/VOQCHQ

17.	Almquist ZW, Helwig NE, You Y. Connecting Continuum of Care point-in-time homeless counts to United States Census areal units. *Math Popul Stud*. 2020 2020;27(1):46-58. doi:10.1080/08898480.2019.1636574

18.	Center for Disease C, Prevention. Data from: Behavioral risk factor surveillance system. 2025.

19.	Center for M, Medicaid S. Mapping medicare disparities (MMD) tool | CMS.

20.	National Center for Hiv VHSTD, Tuberculosis P. About AtlasPlus. *About AtlasPlus | CDC NCHHSTP*. 2025/06/04/T09:14:31Z 2025;

21.	Center for Disease C, Prevention. NVSS - National vital statistics system homepage. 2025/07/23/T11:28:12Z 2025;

22.	Center for Disease C, Prevention. Cdc wonder.

23.	Center for Disease C, Prevention. CDC WISQARS - web-based injury statistics query and reporting system. *Center for Disease Control and Prevention*.

24.	The fed - map: County-level debt-to-income ratio, 1999 - 2025:Q1.

25.	EDFacts.

26.	Data from: State and county dashboard. 2025.
